# Ten simple rules for elevating Indigenous partnerships in research

**DOI:** 10.1371/journal.pcbi.1014770

**Published:** 2026-09-16

**Authors:** Alice Bradley, Emily Lescak, Margaret Rudolf, Brandi Kamermans, Hajo Eicken

**Affiliations:** 1 Geoscience Department, Williams College, Williamstown, Massachusetts, United States of America; 2 International Arctic Research Center, University of Alaska Fairbanks, Fairbanks, Alaska, United States of America; 3 Alfred Wegener Institute Helmholtz Centre for Polar and Marine Research, Bremerhaven, Germany; Carnegie Mellon University, UNITED STATES OF AMERICA

## Introduction

The Research Networking Activity for Sustained Coordinated Observations of Arctic Change (RNA CoObs) aims to create meaningful partnerships with Indigenous Peoples towards the co-design of Arctic observing and monitoring systems and grow a community of practice that serves societal needs while adding shared value for decision-making [1,2]. The project team consists of non-Indigenous academics and five early-career Indigenous Liaisons, in partnership with Alaska Native community members, as well as conference collaboration with Sámi within Europe, Inuit within Canada and Greenland, and Indigenous scholars from Russia. These ten simple rules stem from our experience bridging Alaskan and pan-Arctic contexts and apply decades of recommendations by Indigenous scholars and organizations (e.g., [3,4] and others) to principles of project management.

Indigenous-engaged research exists on a spectrum, from Western science-led projects informed to some degree by Indigenous priorities to Indigenous-led research centered in the methods and worldview of an Indigenous People [5]. We are not the first to publish recommendations for good practice in engaging Indigenous partners in research. The rules we describe are intended for Western scientists making steps towards true co-production of knowledge (defined as equitable collaboration between Indigenous Knowledge Systems and scientific approaches towards new understanding [6]). This goes beyond avoiding “helicopter research” [7] to Indigenous colleagues being equal partners in research within the context of existing funding and academic structures. Movements toward Indigenous Research Sovereignty aim much further: Indigenous-led research, priority setting, and funding decisions [4,8–10].

**Fig 1:**
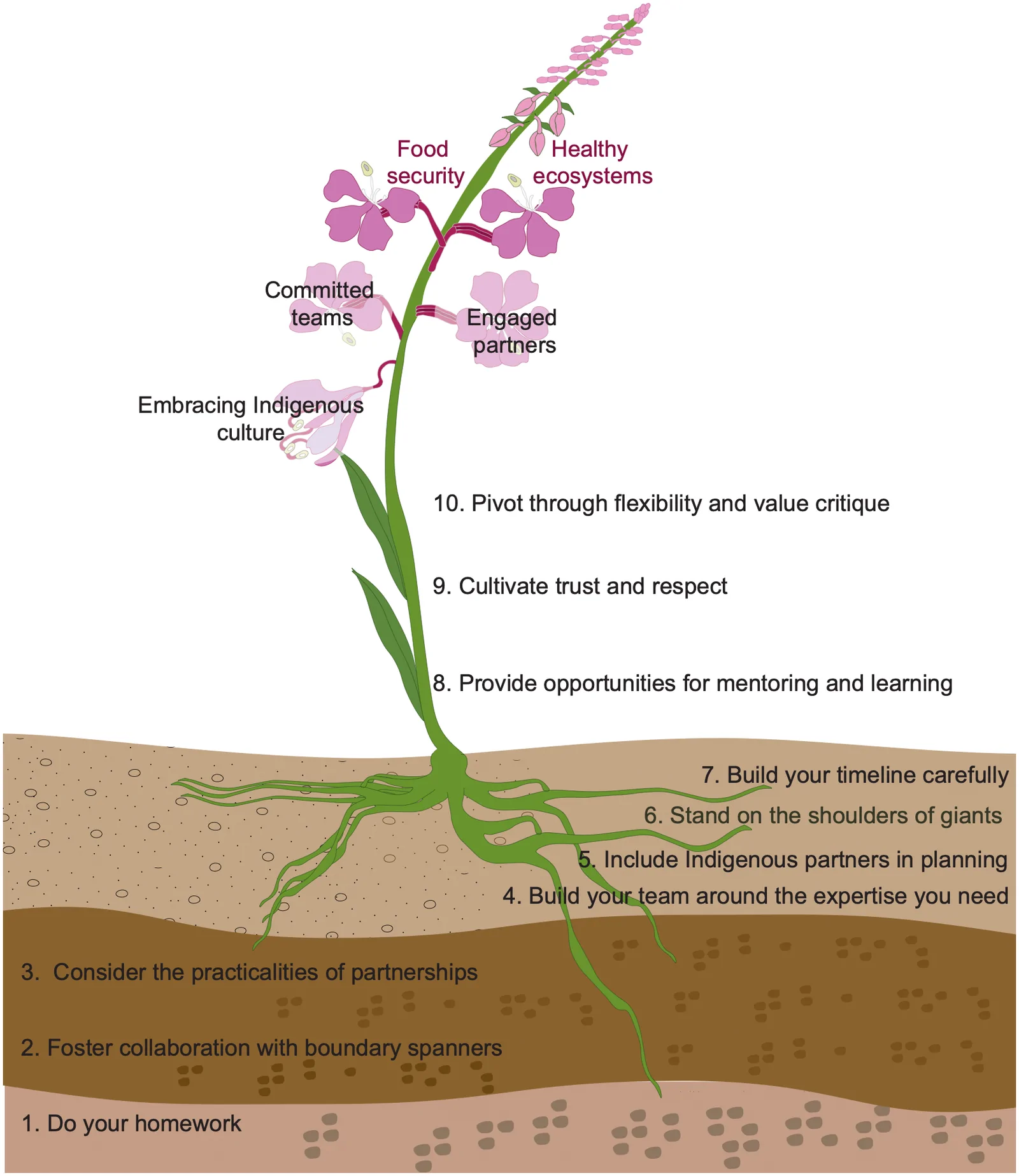
Plants flourish when they have healthy roots in rich soils. Likewise, projects built with strong and healthy root systems are more likely to be fruitful.

Our rules are analogous to growing a plant (Fig 1): the first three prepare the soil, from which the roots form the foundation established during proposal development (4–7) so that the stem (8–10) can support the project throughout its lifetime and allow lasting contributions to bloom.

## Rule 1: Do your homework

Read works by Indigenous scholars, leaders, and organizations (e.g., [3]), take classes (e.g., AlaskaX: Navigating Actionable Science in the Arctic), and participate in trainings (e.g., First Alaskans Institute) to understand the history, cultural context, and governance systems around the work you are proposing to do ([4], call 7). It is your responsibility to understand the required permissions and engagement protocols, often specific to a particular community ([3,4] ([11] calls 1, 7)). Indigenous organizations publish research protocols (e.g., [3,11]), data sovereignty considerations [12], and evaluation frameworks (e.g., [6,13]) that researchers need to adhere to and assess their work against. Be aware of the institutional barriers that can impede co-production with Indigenous partners: funding constraints and timescales, a reluctance in academia to elevate Indigenous Knowledges alongside academically-trained knowledge systems [14], and personnel turnover that can impair relationships between research institutions and communities [15,16]. Seeking out one or more Indigenous mentors (see Rule 8)—if there is mutual interest and resources to support such a relationship—can help, but their role is not to teach the research team about history.

## Rule 2: Foster collaboration with boundary spanners

Diverse teams improve science through the publication of more novel, robust, and high-impact research than homogenous teams ([17] ([18] Rule 1)). Incorporating multiple ways of knowing, or perspectives that are based on an individual’s culture and geography [19,20], has been shown to improve engagement between researchers and Indigenous communities [15].

CoObs utilized boundary spanners [21,22] to bridge gaps between academia, Indigenous communities, and policy decision-making spheres. But don’t rely on them alone—every team member should engage in communication, relationship-building, and social learning to share the responsibility of connecting different knowledge systems. Budget for project management, coordination, and administrative support so that these responsibilities are not disproportionately assumed by the boundary spanner(s). Having both Indigenous and non-Indigenous project team members representing a range of career stages can be beneficial when navigating tensions within a project that may result from the history of suppressing Indigenous Knowledge and practicing colonial science [23,24].

## Rule 3: Consider the practicalities of partnerships

With strategic and well-nurtured partnerships, the potential for both productivity and lasting change can be much greater than what a single group could accomplish. Whether these partners are organizations, projects, or individuals, building and maintaining relationships does not happen overnight or for free. Seed funding, such as the Alaska Climate Adaptation Science Center Ambassador Program, the Burroughs Wellcome Fund Climate and Human Health Seed Grants, and planning grants through the National Science Foundation can support this phase.

Indigenous partners face challenges to their engagement that may not be obvious to scientists. They often have demands on their time including subsistence practices, cultural activities, and community commitments, as well as the need to respond to proposed actions by federal or state agencies affecting the community [25,26]. Their organizations may not have the same level of funding support, and their priorities might be different than yours.

Follow Tribal research protocols (e.g., [3,11]) to understand good practices for engagement. Consider how and where to meet partners: Are you asking them to come to you? Is what you’re doing providing value to them? This is especially important where there might be a real or perceived power difference [27]. The CoObs team held meetings at the Alaska Forum, a conference with significant tribal presence, which helped us build the foundation of community-driven Expert Panels focused on key Arctic variables [2]. Indigenous partners are less likely to have access to travel funding [28], so whenever possible, we provided honoraria and travel support for partners who engaged in our meetings and in conferences relevant to our work (e.g., the Arctic Observing Summit). Inquiring early about what your partners need to fully engage will make a difference in whether these collaborations are mutually beneficial.

## Rule 4: Build your team around the expertise you need

Think critically about what knowledge, skills, and connections you need to achieve project goals. Projects with meaningful Indigenous community participation are much more likely to reflect community values [29], so be sure the staffing reflects that. In CoObs, an Indigenous partner pulled out of the collaboration late in the proposal-writing phase, leaving a gap in expertise within the project team. Plan time into the proposal-writing stage to reach out to new collaborators and build partnerships (Rule 3) to have the full range of expertise on the team. Fund the team proportionally to their importance to the project. If Indigenous participation is core to the goals, make sure a fair fraction of the project budget goes to facilitating that participation (see the supplement for recommended budget categories).

## Rule 5: Include Indigenous partners in planning

What communities are you trying to reach with the goals of the project? What information or products are they looking for? How—in what format—do they want to receive those results? Do not assume you know the answer to that question without asking—this is where building the team around the relevant expertise and connections (Rule 4) is important. The CoObs-supported salmon expert panel decided that workforce development was central to their recommendations for improved salmon monitoring, far outside the original scope of CoObs. To better understand risks associated with increasingly frequent Harmful Algal Blooms, scientists have proposed more scientific research on HABs or early warning systems. Tribes instead requested assistance in risk communication that supports cultural shellfish harvesting while empowering informed decision-making on consumption. It is easy to get the deliverables wrong if you do not engage your partners early enough in the process. Planning ahead is easier than pivoting later (see Rule 10). Actionable science requires knowing who is going to use the results of the project and having their input in every step of the process [30,31]

## Rule 6: Stand on the shoulders of giants

Scientific progress is built on the work of others. This is even more important when working in Indigenous contexts. Where there has been a history of Western institutions disregarding Indigenous experts’ opinions, requests, and needs [32], there is an understandable sensitivity to having prior work ignored. Researchers from outside the Arctic coming in year after year demands time and attention from communities [33], which can be especially frustrating when similar work has been done before. Boundary organizations (e.g., UIC Science), created to mitigate some of the research pressure on Indigenous communities, can be an incredible resource for finding what has been done before and being respectful of potential partners’ time.

## Rule 7: Build your timeline carefully

Once you know what the project is trying to achieve, build a timeline. Plan for relationship-building and the early phases of co-production of knowledge to take a lot of time [34,35]. Schedule around cultural and subsistence activities—nobody is going to be available to talk to researchers during fishing season. Successful co-production happens “at the speed of trust,” and that is not a process that can be rushed. Skipping the building of solid, respectful relationships at the beginning of a project may cause problems later (Rule 5, [35]).

There are often insufficient funds to fully support everyone involved in the project at the maximum level for the full duration, especially for large, collaborative proposals with many partners. It can be a successful strategy to stagger when different efforts, institutions, and participants have more funding. There are two potential pitfalls with this approach: participants in the later phases of the project should be engaged from the beginning to benefit from learning along the way, and there is a risk that project pivots (see Rule 10) could be incompatible with funding allocations over the project’s lifetime. Budgeting for a small amount of participation throughout the project can help with the former, and working with program managers can address the latter.

## Rule 8: Provide opportunities for mentoring and learning

Meaningful collaboration requires intentional investment in workforce development and capacity building, particularly in Indigenous and Arctic communities with limited infrastructure, resources, and access to specialized training. Insufficient training opportunities can prevent full participation from communities [36]. Co-produced research should build sovereign scientific infrastructure that centers Tribal leadership, supports local monitoring, and provides training and workforce development for Tribal members ([4] call 5). At the same time, projects should provide opportunities for mentoring and learning within the project team. In CoObs, the early-career Indigenous liaisons took on heavier leadership burdens without senior Indigenous mentorship within the project. By partnering with faculty in the University of Alaska Fairbanks College of Indigenous Studies as well as other experts, we provided both formal and informal learning opportunities for project team members and partners.

## Rule 9: Cultivate trust and respect

Building a culture of trust and respect among project team members is vital and requires accountability at all levels. We recommend collectively developing agreements or guidelines that describe expectations for interactions, with assistance from a professional facilitator. Helpful resources include The First Alaskans Institute, the CLEAR Lab, and The Art of Gathering [27]. Prioritize times that are most conducive to partners, taking into account Council Meetings, policy-relevant meetings (e.g., State Board of Fisheries), and subsistence activities when planning project activities and deadlines.

A culture of trust extends to data management and information sharing. Develop an understanding of CARE principles (Collective Benefit, Authority to Control, Responsibility, and Ethics [37,38]) and have meaningful conversations about how to implement them (e.g., [39]) before collecting data. Share data and results with relevant communities [40] prior to broad distribution. Data can be stewarded within Tribally-led databases, for example the Indigenous Sentinels Network (ISN) database, owned and operated by the Aleut Community of St. Paul Island Tribal Government, ensuring that community partners retain ownership, governance, and control over data [39].

## Rule 10: Pivot through flexibility and value critique

Changes in project personnel and improved understanding of community needs may require pivots, particularly in long-term projects (see Rule 5 and the need for appropriate deliverables). Feedback from the project team, partners and program managers is critical to adapting. Consider evaluation frameworks (e.g., [5,6]) and other means of soliciting feedback early and throughout the project. Be prepared to take “no” for an answer from potential partners or collaborators. Maintaining a culture of openness helps build trust, respect, and ultimately makes for a more effective project (see ([31] Rule 8)). The CoObs project incurred unexpected delays and challenges due to the pandemic, unanticipated increases in involvement in the planning of the Arctic Observing Summit, piloting of the SAON ROADS process [41], and the project team learning more about the principles of co-production of knowledge [6]. Regular communication with our program manager enabled us to re-evaluate and more clearly define our priorities and deliverables.

## Conclusions

Successful projects that bridge Indigenous and Western scientific approaches and knowledge systems bring together expertise, careful planning, sufficient money and time, and a common goal rooted in a culture of mutual respect. Co-production requires equitable inclusion of everyone’s perspectives from the beginning—setting the direction and goal for the project—through to the conclusion. But how do you get the right people in the room before you know what the project is trying to accomplish? All partners require financial support to participate and their engagement requires funding before determining the direction of the project. Getting out of this loop requires iteration and learning, cultivating relationships from which a project built on mutual trust and respect can grow (Rules 1–3). When approached with care and deliberation, the proposal development process (Rules 4–7) can form strong roots that help the group thrive. Commitment to learning, trust and flexibility (Rules 8–10) builds a stem that supports a project culture ready to bear fruit (Fig 1). In much the same way that funders and publishers accelerated the open science movement by mandating data management and sharing policies, they can also plant the seeds of good practices in ethical co-produced research.

With the need to be concise in our 10 Simple Rules, there are lessons from our project and literature that we are not able to address. Furthermore, these rules are not ‘simple’—following our guidance requires commitment to learning and relationships, iteration in approaches, and a willingness to accept and learn from criticism. Our lens is specific to Alaska and while this advice is broadly applicable across the Arctic, we stress the importance of understanding local context when building new relationships. We encourage researchers to familiarize themselves with frameworks such as Ellam Yua [6] and David-Chavez and Gavin [5] so that they can self-assess their research practices and better understand how to evaluate partnerships through an Indigenous lens.

## Supporting information

S1 Table Table of recommended budget items and their justifications. (PDF)
