# Supplementary material for: Ten simple rules for elevating Indigenous partnerships in research
Source: PLoS Comput Biol. 2026 Sep 16;22(9):e1014770. doi: 10.1371/journal.pcbi.1014770 (PMC13580992; doi:10.1371/journal.pcbi.1014770)
Supplement: Supplementary file 1 [file pcbi.1014770.s001.pdf]

Ten Simple Rules for elevating Indigenous partnerships in research

Supplement

Summary of often-overlooked budget items and their justifications.

**Budget item**	**Justification**
Seed funding for relationship building	Community events, travel to in-person meetings, and time spent engaging with potential project partners is not free. Seed funding and planning grants can cover pre-project costs, especially for Indigenous partners who may not have institutional support for the proposal-writing phase.
Professional development opportunities for project team members across career stages	Opportunities for professional development are critical in the early stages of someone's career, but learning does not stop when someone ages out of the "early career" designation.
Trainings	Some Indigenous organizations provide training sessions that can be a valuable part of project group development. These sessions have costs.
Travel costs for project team members to meet Indigenous partners in their communities or at gatherings that they are planning to attend	Traveling to Indigenous partners (whether their communities or gatherings and conferences they already plan to attend) means that you avoid requiring them to do extra travel. This is a sign of respect for their time.
Honoraria and travel support for partners	Travel for Indigenous partners is often more expensive than projects budget for, between costs of traveling from remote communities, time to adjust to time zones, and additional responsibilities (e.g., childcare).
Equitable funding for Indigenous project team members	Salaries can vary substantially between institutions and between Indigenous and Academic employers. Consider the time allotted to each member of the project team: Indigenous researchers and boundary spanners may need more time because a significant portion of their role is making and maintaining relationships with community partners.
Early participation for project	Learning and slight changes in project direction and culture

team members with more substantial roles later in the project	develop over time. For project team members who are more engaged later in the project, having sufficient resources that they can participate in trainings and meetings throughout means they are prepared to contribute when their time comes.
Project management / coordination and communication support	These responsibilities should not be disproportionately assumed by early career researchers and boundary spanner(s) on the project team.
Expert evaluation services	Project evaluation requires specific expertise. Especially where a project is being evaluated for Indigenous engagement, experience applying Indigenous engagement frameworks is essential.
